# Supplementary material for: Development of lipL32 real-time PCR combined with an internal and extraction control for pathogenic Leptospira detection
Source: PLoS One. 2020 Nov 2;15(11):e0241584. doi: 10.1371/journal.pone.0241584 (PMC7605690; doi:10.1371/journal.pone.0241584)
Supplement: Attachment — Submitted filename: Point wise comments to authors PONE-D-20-04275.docx [file pone.0241584.s001.docx]

**Point wise comment to authors**

***Development of lipL32 real-time PCR combined with an internal and extraction control for pathogenic Leptospira detection***

***Manuscript No: PONE-D-20-04275***

***Abstract:***

1. Page 9, line 127- 128: The basic source of Internal control (IC) is missing. What are the criteria adopted for designing of IC? In which cloning vector used? Is any previous references are available?
2. Page 10, line 136 & 150: Initially it is mentioned that optimization of real time PCR using light cycler 480 probes Master mix (Roche, Germany), later in line 150 2X master mix (Applied Biosystem). Which is exactly used in this study? Kindly modify accordingly
3. Page 11, Line 161 -172: In analytical specificity, 10^3^ genome copies were used. This is equal to 1000 organisms/reaction. Whereas in analytical sensitivity serial dilution (10 fold) of DNA used. Here exact counting of organism is practically difficult. So author may incorporate Initial concentration of DNA/ copy number used for dilution needs to be mentioned, which is missing. Standard curve normalization w.r.t 10^3^ genome copies needs to be incorporated along with the double dilution as well.
4. Page 12, line 175: What is the spiked leptospiral (*L.interrogans* strain Kantorowic) load used in the first dilution of this study?
5. Page 12, Line 186 -189: Mentioned as “positive PCR. Which type of PCR is missing? Also authors are requested to analyze the data from each case inclusion criteria and draw their positive and negative results obtained from this study. I believe these data may provide certain clues where/in which case(s) the diagnostic efficiency are increased. These findings would helpful for the selection of appropriate sample and its day of collection after onset of disease.
6. Line 105 -106: Mentioned as all leptospiral DNA concentration equivalent to 10^3^ genome/µl. whereas in line 154, 10µl of DNA template was used for the amplification. This is approximately equal to 10,000 copies. In line 163, 10^3^ genome copies per reaction were used. These three statements are having contradicted interpretation and confuse the readers. Authors are requested to modify accordingly.
7. Page 13, line 203-204: any organism other than leptospira; modify other than “pathogenic” leptospira.
8. Line 107 & 172 -173: 100 copies of IC DNA was spiked in clinical specimens for determination of efficiency of extraction. Whether authors are achieved same 100 copies in the extraction? This is missing. Line 172 -173: The concentration of IC template was optimized as 50 copies/reaction. In line 214, considering the above statement, IC template should be two copies/reaction, but it is mentioned one copy reaction. Kindly clarify the statement.
9. Page 14, Line 223 -224: Clinical sample ratios (Positives & Negatives) are statically insignificant. More negative samples were enrolled in the study.
