# Supplementary material for: Development of lipL32 real-time PCR combined with an internal and extraction control for pathogenic Leptospira detection
Source: PLoS One. 2020 Nov 2;15(11):e0241584. doi: 10.1371/journal.pone.0241584 (PMC7605690; doi:10.1371/journal.pone.0241584)
Supplement: Attachment — Submitted filename: Rebuttal letter with Point wise comments to authors.docx [file pone.0241584.s002.docx]

Rebuttal Letter	Manuscript No: PONE-D-20-04275, Development of lipL32 real-time PCR combined with an internal and extraction control for pathogenic Leptospira detection

Amsterdam, 26 June 2020

Dear Dr. Kalimuthusamy Natarajaseenivasan,

Thank you very much for the opportunity to revise our manuscript ‘Development of lipL32 real-time PCR combined with an internal and extraction control for pathogenic Leptospira detection’.

Please find below our response.

Yours sincerely,

A.A. Ahmed PhD

**Point wise comment to authors**

***Development of lipL32 real-time PCR combined with an internal and extraction control for pathogenic Leptospira detection***

***Manuscript No: PONE-D-20-04275***

***Abstract:***

1. Page 9, line 127- 128: The basic source of Internal control (IC) is missing. What are the criteria adopted for designing of IC? In which cloning vector used? Is any previous references are available?

Answer:

This is synthetic DNA, we will change in the text “artificial” to “synthetic”. The criteria are that the sequence of the synthetic DNA does not react with the used target primers sequences and that the primers used to amplify the IC should have an annealing temperature equalling 60 °C as for the target primers. The cloning vector is pUC57-Kan. We have added this information to the manuscript. There is no previous reference available, the IC is designed for the first time described in this manuscript.

2. Page 10, line 136 & 150: Initially it is mentioned that optimization of real time PCR using light cycler 480 probes Master mix (Roche, Germany), later in line 150 2X master mix (Applied Biosystem). Which is exactly used in this study? Kindly modify accordingly.

Answer:

Thank you for this remark, we used Roche, Germany for both and we have modified the text accordingly.

3. Page 11, Line 161 -172: In analytical specificity, 10^3^ genome copies were used. This is equal to 1000 organisms/reaction. Whereas in analytical sensitivity serial dilution (10 fold) of DNA used. Here exact counting of organism is practically difficult. So author may incorporate Initial concentration of DNA/ copy number used for dilution needs to be mentioned, which is missing. Standard curve normalization w.r.t 10^3^ genome copies needs to be incorporated along with the double dilution as well.

Answer:

Initial concentration of DNA has been added to the MS (i.e. strain Kantorowicz genomic DNA starting at 1 × 10^4^ copies per reaction). The standard curve with 10-fold dilution is added to the manuscript as Figure 1. We did not perform a standard curve with doubling dilution, though the limit of detection is mentioned already in the text.

4. Page 12, line 175: What is the spiked leptospiral (*L.interrogans* strain Kantorowicz) load used in the first dilution of this study?

Answer:

The load of the strain Kantorowicz species *L. interrogans* (9x10^5^ copies) of used in the first dilution has been added to the text as well as the volumes of blood, serum and urine.

5. Page 12, Line 186 -189: Mentioned as “positive PCR. Which type of PCR is missing?

Also authors are requested to analyze the data from each case inclusion criteria and draw their positive and negative results obtained from this study. I believe these data may provide certain clues where/in which case(s) the diagnostic efficiency are increased. These findings would helpful for the selection of appropriate sample and its day of collection after onset of disease.

Answer 1:

The PCR is the secY real-time PCR mentioned in Reference 3, we made it more clear in the manuscript.

Answer 2:

Thank you for your suggestion to analyse the data from each case based on the inclusion criteria, in order to provide possible clues for increased diagnostic efficiency. In our opinion, we might not able to provide these clues. Our case definition is based on the overall results from each patient (serology, culture and secY real-time PCR). We encourage clinicians to submit the appropriate samples according to the days post onset, i.e. 1-10 DPO serum (for serology and PCR), EDTA blood (for PCR) and heparinised blood (for culture), >10 DPO serum (for serology). Urine and CSF can be send at any time point for PCR. However there are limitations to prepare such an analysis as you suggested since the reality is that we often get sub-optimal samples. In this study we also choose to include from each patient only one specimen to perform *lipL32* real-time PCR, while the secY real-time PCR is performed on all submitted samples from the patient eligible for PCR (i.e. serum/EDTA blood taken 1-10 DPO, urine and CSF at any timepoint).

In earlier studies it is shown that PCR on blood samples is most useful during the first 10 days of disease (Esteves LM et al. 2018. Diagnosis of Human Leptospirosis in a Clinical Setting: Real-Time PCR High Resolution Melting Analysis for Detection of Leptospira at the Onset of Disease. Sci Rep. 2018;8(1); Ahmed A et al 2009. Development and validation of a real-time PCR for detection of pathogenic leptospira species in clinical materials. PLoS One. 2009;4(9)).

6. Line 105 -106: Mentioned as all leptospiral DNA concentration equivalent to 10^3^ genome/µl. whereas in line 154, 10µl of DNA template was used for the amplification. This is approximately equal to 10,000 copies. In line 163, 10^3^ genome copies per reaction were used. These three statements are having contradicted interpretation and confuse the readers. Authors are requested to modify accordingly.

Answer:

Thank you for this observation, this is now modified to a concentration equivalent to 10^2^ genome/µl in line 105-106.

7. Page 13, line 203-204: any organism other than leptospira; modify other than “pathogenic” leptospira.

Answer:

this is adapted

8. Line 107 & 172 -173: 100 copies of IC DNA was spiked in clinical specimens for determination of efficiency of extraction. Whether authors are achieved same 100 copies in the extraction? This is missing. Line 172 -173: The concentration of IC template was optimized as 50 copies/reaction. In line 214, considering the above statement, IC template should be two copies/reaction, but it is mentioned one copy reaction. Kindly clarify the statement.

Answer:

We have added an extra paragraph “Efficiency of the extraction method” showing the results of the recovery efficiency when we use 100 copies of IC. In Line 172-173 we wrote the optimal concentration of IC template was 50 copies/reaction. This is implemented when testing clinical samples as well as spiked material. We have rewritten these lines to make it more clear that there is no relation between the 50 copies/reaction used for the clinical samples and spiked material and the 100 copies/reaction used to estimate the extraction efficiency.

9. Page 14, Line 223 -224: Clinical sample ratios (Positives & Negatives) are statically insignificant. More negative samples were enrolled in the study.

Answer

Indeed, more negative patients are enrolled in this study. This reflects the pattern of submitted samples to our diagnostic laboratory, so therefore this is a representative population for the Dutch situation.

**Points of reviewer#2:**

Regarding the statistical method: We have adapted the text, we added the number of cases with a positive *lip*L32 real-time PCR result for both laboratory confirmed leptospirosis cases and negative cases.

We feel this is sufficient for the reader to get insight which proportion of the laboratory confirmed leptospirosis cases as well as negative cases have a positive / negative *lip*L32 real-time PCR result.

We had made already reference to the website < <http://vassarstats.net/>>, we used as a tool for the statistical computation: Clinical Research Calculators/Calculator 1.

Here the 95% confidence intervals for proportions are calculated according to the efficient-score method (corrected for continuity) described by Robert Newcombe, based on the procedure outlined by E. B. Wilson in 1927.

We have adapted the grammar and spelling errors according to the remarks.

**Points of Academic Editor:**

This is addressed above, see reviewer#2, regarding the statistical method.
