# Supplementary material for: Development of lipL32 real-time PCR combined with an internal and extraction control for pathogenic Leptospira detection
Source: PLoS One. 2020 Nov 2;15(11):e0241584. doi: 10.1371/journal.pone.0241584 (PMC7605690; doi:10.1371/journal.pone.0241584)
Supplement: Attachment — Submitted filename: Point wise comments to authors for revision PONE-D-20-04275R1.docx [file pone.0241584.s003.docx]

**Point wise comment to authors for final revision**

***Development of lipL32 real-time PCR combined with an internal and extraction control for pathogenic Leptospira detection***

***Manuscript No: PONE-D-20-04275R1***

Page 11; line 170 -171 (Authors are requested to incorporate ): To assess the effects of biological matrix...........sero negative (MAT) and PCR negative blood and serum samples & PCR negative urine samples were spiked with *L. interrogans* serovar Icterohaemorrhagiae strain Kantorowicz.

Page 14; Line 227 -229: Efficiency of extraction method- Although the spiking was done with 10 fold dilution of 9 X10^5^ leptospires. However, the result mentioned about 100 copies IC only. Kindly clarify/ modify accordingly.

Pgae14: Line 234: Among 71 positive samples, how many paired samples incorporated in this study from the banked samples?

As mentioned in the line 189, the paired samples should be collected at least 2 days apart. Kindly change the word into 2 weeks. (Refer WHO guidelines)

(https://www.who.int/zoonoses/diseases/Leptospirosissurveillance.pdf?ua=1)
