# Supplementary material for: Development of lipL32 real-time PCR combined with an internal and extraction control for pathogenic Leptospira detection
Source: PLoS One. 2020 Nov 2;15(11):e0241584. doi: 10.1371/journal.pone.0241584 (PMC7605690; doi:10.1371/journal.pone.0241584)
Supplement: Attachment — Submitted filename: Response to Reviewers.docx [file pone.0241584.s004.docx]

Rebuttal Letter	Manuscript No: PONE-D-20-04275R1

Development of lipL32 real-time PCR combined with an internal and extraction control for pathogenic *Leptospira* detection

Amsterdam, 25 September 2020

Dear Dr. Kalimuthusamy Natarajaseenivasan,

Thank you very much for the opportunity to adapt our manuscript ‘Development of lipL32 real-time PCR combined with an internal and extraction control for pathogenic Leptospira detection’ with the last suggestions.

Please find below our response.

Yours sincerely,

A.A. Ahmed PhD

- Page 11; line 170 -171 (Authors are requested to incorporate ): To assess the effects of biological matrix...........sero negative (MAT) and PCR negative blood and serum samples & PCR negative urine samples were spiked with L. interrogans serovar Icterohaemorrhagiae strain Kantorowicz.

We have incorporated as requested

- Page 14; Line 227 -229: Efficiency of extraction method- Although the spiking was done with 10 fold dilution of 9 X10^5^ leptospires. However, the result mentioned about 100 copies IC only. Kindly clarify/ modify accordingly.

To clarify: in Material and Methods, page 8 line 100-102 we describe how the efficiency of the extraction method is performed and indeed we used an equivalent of 100 copies of IC DNA and not the 10 fold dilution of 9 X10^5^ leptospires.

- Page14: Line 234: Among 71 positive samples, how many paired samples incorporated in this study from the banked samples?

There were no paired samples included in the 71 positive samples.

- As mentioned in the line 189, the paired samples should be collected at least 2 days apart. Kindly change the word into 2 weeks. (Refer WHO guidelines). (<https://www.who.int/zoonoses/diseases/Leptospirosissurveillance.pdf?ua=1>)

Thank you for your remark. Indeed, the WHO guidelines mention to collect paired samples at least 2 weeks apart. However in our laboratory (National Leptospirosis Reference Laboratory (NRL)) we found that a titre rise can even be observed in samples 2 days apart. This is described in the reference [2], already cited in line 185. To clarify, we have cited this reference again at the end of line 189.
